# Local Control and Vertebral Compression Fractures After Stereotactic Body Radiotherapy for Spinal Metastases

**DOI:** 10.3390/jcm14217718

**Published:** 2025-10-30

**Authors:** Ha Un Kim, Jinhong Jung, Young Seok Kim, Yeon Joo Kim, Young Seob Shin, Su Ssan Kim

**Affiliations:** Department of Radiation Oncology, Asan Medical Center, University of Ulsan College of Medicine, 88 Olympic-ro 43-gil, Songpa-gu, Seoul 05505, Republic of Korea; d210194@amc.seoul.kr (H.U.K.);

**Keywords:** spine, metastasis, stereotactic body radiotherapy, vertebral compression fracture

## Abstract

**Objectives:** This study aimed to evaluate the efficacy and toxicity of stereotactic body radiotherapy (SBRT) for spinal metastases, focusing on pain control, local tumor control, and the incidence of vertebral compression fractures (VCF). **Materials and Methods:** We retrospectively analyzed 179 patients with 217 spinal metastatic lesions who underwent SBRT between July 2020 and April 2022. The prescribed doses for SBRT were 18 or 20 Gy for one fraction, ≥24 Gy for three fractions, ≥20 Gy for four fractions, and ≥25 Gy for five fractions. Patient-reported treatment response was evaluated 1–3 months after SBRT completion. Local recurrence was defined as failure within the radiotherapy field. Pain response, local progression-free survival (LPFS), and the incidence of painful VCF were assessed. Prognostic factors for LPFS and VCF risk factors were evaluated. **Results:** The overall pain response rate was 80.8%. LPFS rates were 90.6% at 1 year and 83.0% at 2 years. Lytic/mixed lesions and involvement of multiple segments were significant prognostic factors for reduced LPFS. The cumulative incidence of painful VCF was 8.7% at 1 year and 12.8% at 2 years. A biologically effective dose (BED_3_) ≥104 Gy was the only significant risk factor for painful VCF. **Conclusions:** SBRT demonstrated high efficacy for pain and local tumor control in spinal metastases, with an acceptable VCF risk.

## 1. Introduction

Spinal metastasis is one of the frequent sites of tumor spread, occurring in approximately 50–70% of patients with cancer [1,2,3]. Among patients with spinal metastases, 20% exhibit symptoms that can result in serious complications, such as compression fractures or spinal cord compression [4]. The initial approach for managing patients with spinal metastases is to determine whether surgical intervention is required. For those not requiring surgery, conventional external beam radiotherapy (CRT) has been the standard treatment to relieve pain, improve or maintain neurological function, and achieve tumor control.

Recently, advances in targeted therapy, hormone-targeting drugs, and immune checkpoint inhibitors have improved long-term overall survival [5,6]. The prolonged survival has raised the importance of maintaining long-term local control of spinal metastases. In this context, stereotactic body radiotherapy (SBRT) offers several advantages over CRT, delivering high biologically effective doses (BED) of radiation to tumors while minimizing toxicities to surrounding normal tissues in a few fractions (typically 1–5). The efficacy of SBRT has been particularly notable in metastatic diseases with traditionally radioresistant histologies, such as renal cell carcinoma, hepatocellular carcinoma, and sarcoma [7,8]. Specifically in spinal metastases, SBRT has demonstrated superior outcomes compared to CRT, with high rates of tumor control (80–90%) and low rates of toxicity [9,10,11]. Large studies have reported overall and complete pain response rates of approximately 82% and 43.5%, respectively [12], and various SBRT doses and fractionation schemes were used in these studies. Large prospective studies have reported doses ranging from 16 or 20 Gy in a single fraction to 24 Gy in two fractions, 30 Gy in three fractions, or 35 Gy in five fractions [13].

However, vertebral compression fracture (VCF) is a potential late adverse effect of SBRT, causing pain and spinal instability, sometimes requiring stabilization or decompression. VCF rates are reported to be 6–14% [14]. Predictive factors for VCF have been identified including radiographic tumor features (e.g., lytic lesions or baseline VCF), patient characteristics (e.g., sex and age), and radiotherapy dose and fractionation (e.g., dose per fraction ≥ 20 Gy).

This study aims to assess patient-reported pain relief and evaluate the efficacy and incidence of VCF in patients with spinal metastases treated with SBRT at a large tertiary medical center in Korea.

## 2. Materials and Methods

We retrospectively reviewed the medical records of patients who underwent spinal SBRT at a single institution from July 2020 to April 2022. The inclusion criteria were as follows: (1) spinal metastasis confirmed by computed tomography (CT), magnetic resonance imaging (MRI), or positron emission tomography/CT (PET/CT); (2) age ≥ 18 years; and (3) Eastern Cooperative Oncology Group (ECOG) performance status of 0–2. Patients with previous surgery or radiotherapy to the involved vertebral region were excluded. Patient selection was based on our institutional guidelines, which recommend SBRT as primary treatment for patients with spinal metastases who have SINS scores < 7 (stable spine) and no or minimal neurological symptoms. In selected asymptomatic patients, SBRT was also offered to prevent progression that could lead to future pain or neurological compromise, to treat lesions with concerning radiographic features despite absence of symptoms, or according to institutional protocols recommending upfront SBRT for radioresistant histologies. Patients with SINS scores ≥ 13 or ESCC grade 3 requiring surgical intervention before radiotherapy were excluded from this study. Patients with multiple myeloma and other hematopoietic malignancies were also excluded, as these malignancies exhibit different radiosensitivity characteristics and patterns of spinal stability compared to solid tumors. A total of 179 patients were included in the analysis, with 217 treated lesions. Radiosensitive histology was defined as breast and prostate cancer. Radioresistant histology was defined as all other cancer types.

Before radiotherapy, the patients underwent a neurological examination and pain assessment. CT simulation was performed with a 2.5 mm slice thickness. Gross tumor volume was delineated using CT, MRI, or PET/CT, and clinical target volume was set by the radiation oncologist, considering tumor volume and organs at risk. Target volume doses were prescribed to be ≥95% of the radiotherapy dose. The selection of dose and fractionation was determined by a radiation oncologist, considering the characteristics of the spinal lesion and the dose constraints of the surrounding organs. The selection of dose and fractionation was determined by radiation oncologists based on several factors including distance to the spinal cord, tumor histology and size, ECOG, and history of previous radiotherapy. Based on these considerations, the schedule consisted of one to five fractions, with prescribed doses of 18 or 20 Gy for one fraction, ≥24 Gy for three fractions, ≥20 Gy for four fractions, and ≥25 Gy for five fractions. Simultaneous integrated boost (SIB) was permitted.

Patient-reported treatment response was evaluated 1–3 months after SBRT completion through medical records of follow-up visits and analgesic medications. Pain response was categorized as complete response (CR) (patients reporting being pain-free during follow-up visits), partial response (PR) (patients reporting feeling “less pain” compared to baseline, or documented reduction in analgesic medication dosage or frequency without increased pain), stable pain (patients reporting pain levels as “unchanged” or “the same” as baseline with stable medication requirements), or progressive pain (patients reporting worsening pain or requiring increased analgesic medication dosage). Only patients who provided clear, documented responses regarding their pain status during follow-up visits were included in the pain analysis. Patients without explicit pain-related reports or those with ambiguous responses were excluded from the pain assessment.

Radiologic response was assessed using CT, MRI, or PET/CT and evaluated according to the Response Evaluation Criteria in Solid Tumors (RECIST) ver. 1.1 and European Organization for Research and Treatment of Cancer PET criteria [15,16]. Local recurrence was defined as failure within the radiotherapy field. Local progression-free survival (LPFS) was calculated from the completion of radiotherapy to local recurrence.

Toxicity was evaluated according to the Common Terminology Criteria for Adverse Events (version 5.0). A painful VCF identified during a follow-up examination was defined as a new endplate fracture or progression of collapse deformity compared with the pre-SBRT imaging findings.

Survival curves for LPFS were constructed using the Kaplan–Meier method. All reported *p*-values are two-sided, and the significance level was set at 0.05 for all analyses. Cox and logistic regression analyses were used to determine the prognostic and risk factors for LRFS and painful VCF. Prognostic factors with *p*-values less than 0.1 were included in the multivariable analysis. All statistical analyses were performed using SPSS software, version 20.0 (IBM, Armonk, NY, USA).

## 3. Results

### 3.1. Characteristics of the Patients and Spinal Lesions

The baseline characteristics of the patients and lesions are summarized in Table 1 and Table 2, respectively. The median age of all patients was 63 years, and the patients were predominantly male. The ECOG performance status was predominantly classified as 1. Among all treated lesions, 9.2% were cervical, 42.9% thoracic, 40.1% lumbar, and 7.8% sacral. The primary tumor histology included lung, prostate, gastrointestinal, breast, and others, in descending order of prevalence. Most lesions exhibited lytic characteristics (57.1%), were treated in a single segment (60.0%), and were documented to have pre-existing VCF based on MRI or CT scans before SBRT (25.8%). More than half of the lesions (76.5%) were symptomatic. Table 3 summarizes the dosimetry values and their associated characteristics. More than half of the lesions were treated with five fractions (66.4%) and SIB (58.5%). The median gross tumor volume and prescribed BED_10_ were 19.3 mL (range, 1.1–521.0) and 48.0 (range, 30.0–72.0), respectively.

### 3.2. Response to SBRT

The median follow-up period for the entire cohort was 15.3 months (range, 3.0–43.8). The outcomes of SBRT on the pain associated with each lesion are shown in Table 4. Among all lesions, 166 initially presenting with pain were evaluated for pain response to SBRT, excluding 51 lesions that did not present with pain. Sixty-one lesions (36.7%) achieved CR, and 96 lesions experienced PR.

**Figure 1:**
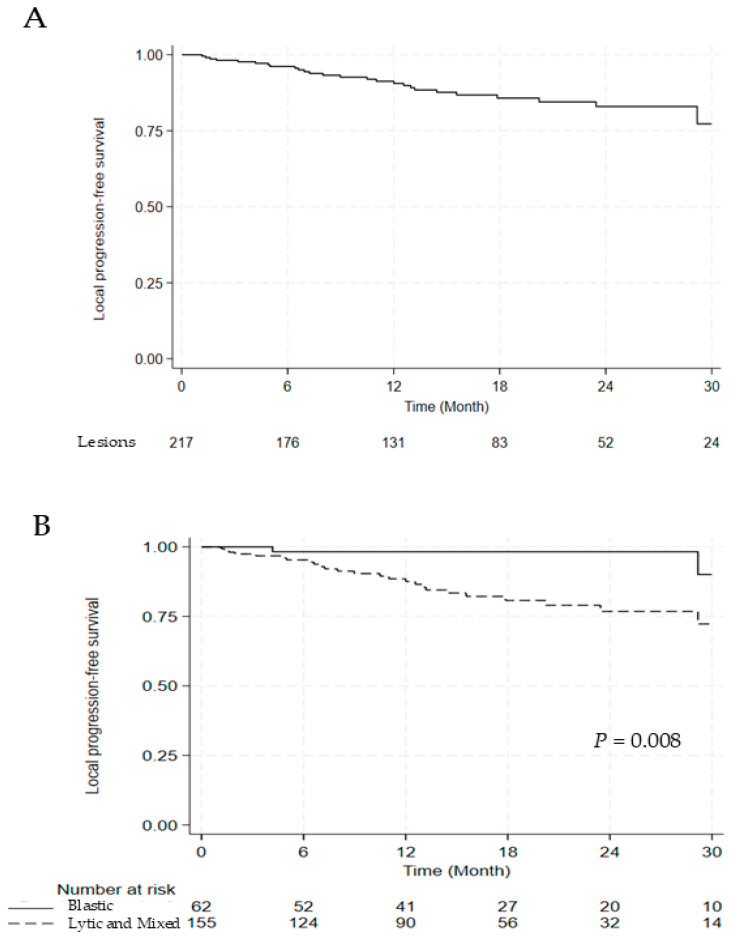

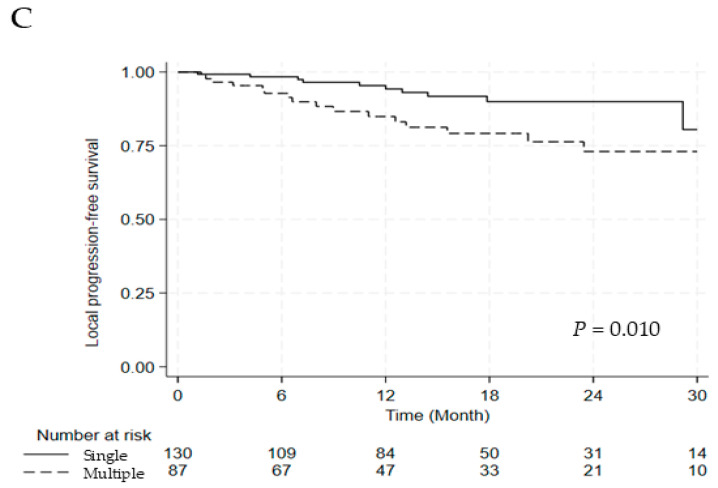
Kaplan–Meier Curve for (**A**) LPFS, (**B**) LPFS According to the Lesion Type, and (**C**) LPFS According to the Segment Number. Abbreviations: LPFS, local progression free survival.

Among 217 lesions, 27 (12.4%) showed local recurrence at the irradiated sites. Of the 27 lesions with local recurrence, 13 (48.1%) occurred within the vertebral body, 2 (7.4%) within the epidural space, and 12 (44.4%) involved both sites. The cumulative incidence of local progression was 9.4% and 17.0% at 1 and 2 years, respectively (Figure 1A). Among these, five patients developed neurological deficits as a result of local tumor progression.

The univariate and multivariate analyses for LPFS are discussed in Table 5. Compared with blastic lesions, lytic and mixed lesions were associated with significantly worse LPFS (hazard ratio [HR] 5.704, 95% confidence interval [CI] 1.349–24.119, *p* = 0.018), and involvement of multiple segments was also an adverse prognostic factor (HR 2.686, 95% CI 1.228–5.874, *p* = 0.013). Significant differences in LPFS were observed between blastic versus lytic and mixed lesions and single versus multiple segments (2-year LPFS: 98.3% vs. 76.8%, *p* = 0.008; 2-year LPFS: 90.0% vs. 73.0%, *p* = 0.010) (Figure 1B,C).

Acute toxicity was evaluated during and within 3 months of SBRT. Acute radiation-induced side effects were grade 1 or 2, including esophagitis (*n* = 2, 1.0%) and diarrhea (*n* = 2, 1.0%). No grade 3 or higher acute toxicities were reported.

### 3.3. Painful VCF

**Figure 2:**
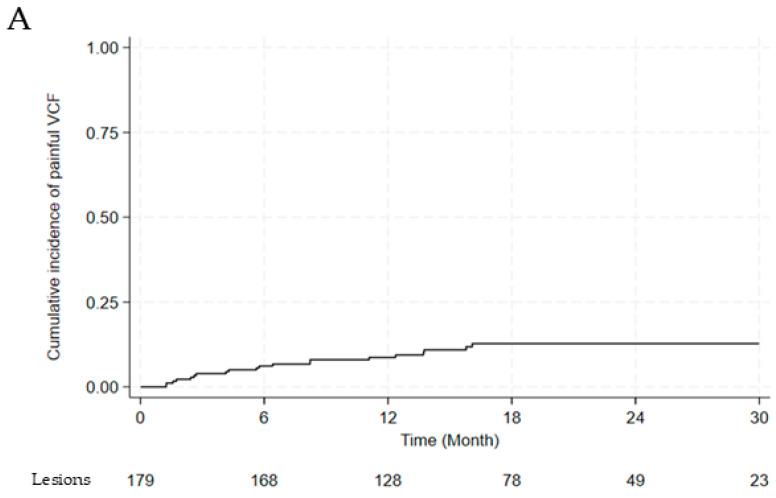

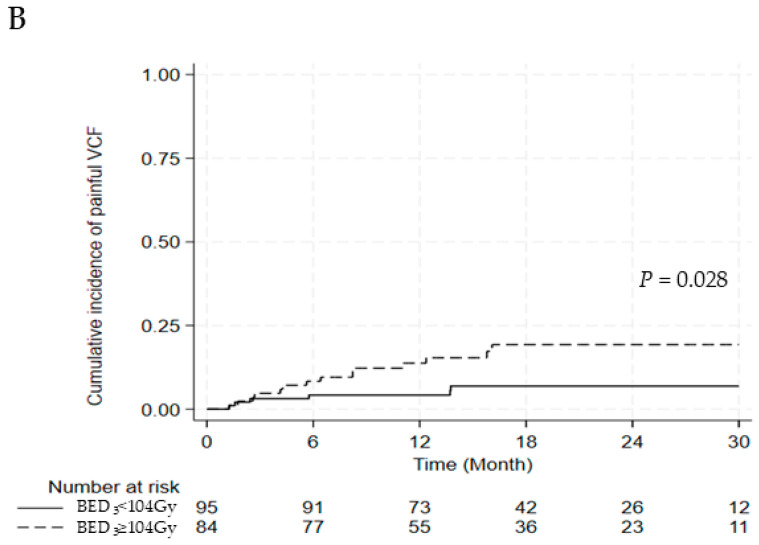
(**A**) Cumulative incidence of painful vertebral compression fractures (**B**) between BED_3_ ≥104 Gy and <104 Gy. Abbreviations: VCF, vertebral compression fracture; BED, biologically effective dose.

We analyzed the incidence of VCF, as shown in Figure 2. One hundred and seventy-nine lesions with a follow-up duration of at least six months were evaluated for the incidence of painful VCF. Twenty painful VCFs were noted among the 179 lesions. The cumulative incidence of painful VCF was 8.7% at 1 year and 12.8% at 2 years (Figure 2A). The incidence of painful VCF stratified by fractionation scheme is summarized in Table 6. The VCF rate for single fraction (*n* = 21) was 19.0% (95% CI: 7.7–40.0). For multi-fraction regimens, rates were 0% (95% CI: 0–39.0) for 3 fractions (*n* = 6), 15.2% (95% CI: 6.7–30.9) for 4 fractions (*n* = 33), and 9.2% (95% CI: 5.2–15.8) for 5 fractions (*n* = 119). While sample sizes were substantially uneven across groups, with 66.5% of evaluable lesions receiving 5 fractions, there was no statistically significant difference in VCF incidence among fractionation schemes (*p* = 0.232).

Six lesions were treated with surgery for spinal stability, three lesions were managed with thoracolumbosacral orthosis, and 11 lesions were controlled with pain medications. Among those who underwent surgery, three patients experienced neurological deficits attributable to vertebral compression fractures. After univariate and multivariate analyses for painful VCF, BED_3_ ≥104 Gy was the only significant risk factor (OR, 2.915; 95% CI, 1.045–8.132; *p* = 0.041) (Table 7). A significant difference was observed in the cumulative incidence of painful VCF between the BED_3_ ≥104 Gy and BED_3_ <104 Gy groups (2-year cumulative incidence of painful VCF: 19.3% vs. 6.9%, *p* = 0.028) (Figure 2A,B).

### 3.4. Subgroup Analysis

Subgroup analysis comparing symptomatic and asymptomatic lesions at baseline was performed to evaluate outcome differences by symptom status. Kaplan–Meier analysis showed a non-significant trend toward better local control in asymptomatic lesions (*p* = 0.093, log-rank test). One-year and two-year LPFS were 97.4% (95% CI, 82.8–99.6) and 92.9% (95% CI, 73.5–98.3) for asymptomatic lesions, versus 86.6% (95% CI, 79.4–91.4) and 80.0% (95% CI, 70.5–86.7) for symptomatic lesions. Local recurrence occurred in 3 of 51 asymptomatic lesions (5.9%) and 24 of 166 symptomatic lesions (14.5%). Among 179 lesions with ≥6 months of follow-up, painful VCF incidence was comparable between groups—4 of 45 (8.9%) versus 16 of 134 (11.9%) (*p* = 0.574).

### 3.5. Overall Survival

The median survival time was 17.3 months (range, 3.0–39.6), and the 1- and 2-year overall survival rates were 65.4% and 36.4%, respectively.

## 4. Discussion

This retrospective study demonstrated that SBRT is a highly effective treatment modality for managing pain and achieving local tumor control in patients with spinal metastases, with acceptable toxicity. The overall pain response rate to SBRT was 80.8%, which aligns with the evolving evidence base supporting SBRT for symptomatic spinal metastases. Early prospective evidence established SBRT efficacy, with Gerszen et al. reporting an overall pain response rate of 86% in one of the largest single-institution series of 500 cases [17]. Subsequently, higher-level evidence from the randomized controlled trials has confirmed SBRT’s superiority. The landmark Canadian Cancer Trials Group Symptom Control 24 (CCTG SC.24) phase II/III clinical trial directly compared SBRT (24 Gy in 2 fractions) to CRT (20 Gy in 5 fractions) in 114 patients, demonstrating a complete pain response rate of 35% at 3 months with SBRT and establishing it as superior to conventional approaches for symptomatic disease [18]. A recent systematic review and meta-analysis by Guninski et al., conducted as part of the 2024 ESTRO practice guideline development, synthesized data from multiple studies and confirmed overall and complete pain response rates of approximately 83% and 36.5%, respectively, establishing contemporary benchmarks for SBRT efficacy [12]. Collectively, these data support SBRT as the preferred treatment modality for painful spinal metastases, and our results corroborate these findings in a real-world clinical setting.

Despite 75.6% of the lesions being classified as radioresistant, our study showed LPFS rates of 90.6% at 1 year and 83.0% at 2 years, consistent with previous studies reporting high tumor control rates (80–90%) with SBRT. These results compare favorably with CRT outcomes, with multiple studies now establishing SBRT’s superiority for local control. The comparative study by Zeng et al. reported significantly lower local failure rates with SBRT versus CRT: 6.1% versus 28.4% at 12 months and 14.8% versus 35.6% at 24 months (*p* < 0.001) in a mature comparative analysis [19]. The CCTG SC.24 randomized trial similarly demonstrated improved local control with SBRT in their head-to-head comparison [18]. Our observed local recurrence rates of 9.4% at 1 year and 17.0% at 2 years with SBRT align with these findings and fall within the range reported by the systematic review by Guckenberger et al., which synthesized available evidence and reported local control rates of 80–95% at 1–2 years with SBRT, substantially higher than historical CRT [20]. However, several population-specific factors warrant consideration when interpreting these outcomes. The histologic composition of our cohort—mainly lung, prostate, and gastrointestinal cancers—may differ from other series, affecting the generalizability of local control estimates. The pronounced male predominance (74.9%), largely due to prostate and lung cancers, could have influenced lesion characteristics and treatment responses, as osteoblastic metastases from prostate cancer typically show better local control. In addition, the limited median overall survival (17.3 months) introduces competing mortality risk, whereby late recurrences may remain undetected. These factors should be considered when comparing our results with other studies. Optimal dose-fractionation for spinal SBRT remains an area of active investigation, with emerging evidence supporting higher biologically effective doses. The 2024 ESTRO clinical practice guideline, based on comprehensive systematic review, recommends a BED_10_ exceeding 50 Gy for durable local control, equivalent to a single fraction of 18 Gy or 30 Gy in three fractions [20]. This recommendation is supported by the randomized phase III trial by Zelefsky et al., which compared single-fraction 24 Gy versus fractionated 27 Gy in three sessions for oligometastatic disease, including 56% with spinal metastases, and demonstrated superior local control with the higher single-dose approach [21]. Long-term data from the SABR-COMET trial further support the efficacy of ablative doses in oligometastatic settings [13].

However, our analysis did not identify a significant dose–response relationship (no significant difference in LPFS between BED_10_ ≥ 50 Gy and <50 Gy groups). This discrepancy warrants interpretation in the context of our population characteristics. First, our patient cohort included a higher proportion of multiple segments treatments (40.0% vs. single segment), which have been independently associated with reduced local control regardless of dose. Second, other factors, such as tumor histology or concurrent systemic therapies, may influence local control independent of the radiation dose. Additionally, the heterogeneity in the fractionation schedules used in our cohort might have affected the BED calculations and subsequent analysis. Further investigation into these potential confounding factors is warranted to better understand the dose–response relationship in this setting and to optimize SBRT regimens for individual patients. Our analysis identified lytic/mixed lesion characteristics and the involvement of multiple segments as significant prognostic factors for reduced LPFS. Similarly, Guckenberger et al. reported that treating >1 vertebra with SBRT correlated with poor local control (*p* = 0.04; HR, 0.62) in 387 spinal metastases [22]. Osteolytic lesions are known to exhibit more aggressive clinical features than osteoblastic lesions, often causing bone destruction and leading to mechanical instability of the spine. When we analyzed the histologies of lytic, mixed, and blastic lesions, more than half of the blastic lesions had primary tumors in the prostate and breast, while the lytic and mixed lesions were observed in the lung, gastrointestinal tract, and kidney. This heterogeneity in histology might have been attributed to the differences in local control.

Our subgroup analysis suggested modest differences in treatment outcomes according to baseline symptom status. Asymptomatic lesions showed marginally better local control than symptomatic lesions, with 2-year LPFS rates of 92.9% versus 80.0% (*p* = 0.093) and lower recurrence rates (5.9% vs. 14.5%). Although not statistically significant, this trend suggests that earlier intervention before symptom onset may be beneficial in carefully selected patients. The comparable incidence of painful VCF (8.9% vs. 11.9%, *p* = 0.574) indicates that prophylactic SBRT does not increase fracture risk. These findings support selective SBRT for asymptomatic lesions, particularly in patients with radioresistant histology or progressive disease despite systemic therapy, while underscoring the need for prospective validation.

Although the toxicity of SBRT for spinal metastases is generally considered acceptable, VCF is the most common adverse event, often resulting in significant pain and spinal deformity. Higher radiation doses can improve local control, but they increase the risk of VCFs, particularly with single-fraction schedules such as 1 × 24 Gy. We observed cumulative incidences of painful VCF of 8.7% at 1 year and 12.8% at 2 years, falling toward the lower end of the 6–14% range reported in the literature [14]. Several treatment-related factors may account for this relatively favorable toxicity outcome. Our predominant use of multi-fraction regimens likely allowed normal tissue repair between fractions. Additionally, bone-modifying agents were used in 20.7% of patients, which may have provided skeletal protection, particularly among high-risk individuals. Third, SIB was applied in 58.5% of lesions, potentially reducing vertebral body dose by concentrating radiation within the gross tumor volume. However, several potential sources of bias should be considered. The pronounced male predominance (74.9%), largely due to prostate (21.2%) and lung cancers (28.6%), may have attenuated the observed VCF incidence, as female sex is a known risk factor owing to lower bone mineral density and estrogen deficiency. Moreover, the limited median OS of 17.3 months introduces competing mortality risk, whereby late VCFs may remain undetected, likely leading to underestimation of the true cumulative incidence, particularly in longer-surviving populations.

Multiple risk factors for VCF following SBRT have been identified, including patient-related factors (SINS score, baseline fracture, lytic lesions, female sex) and treatment-related factors (dose per fraction, total dose) [14,19,20,22]. The 2024 ESTRO guideline confirmed dose per fraction ≥ 20 Gy as a significant predictor alongside patient-specific factors [20]. Our finding that BED_3_ ≥ 104 Gy was the only significant risk factor for painful VCF (HR 2.915, 95% CI 1.045–8.132, *p* = 0.041) aligns with prior dose-dependent VCF risk studies. The lack of association with other commonly reported risk factors may reflect our predominantly multi-fraction approach (88.5% received ≥ 3 fractions) and male-predominant population (74.9%). These findings underscore the importance of balancing tumor control against VCF risk through appropriate dose-fractionation selection.

Our study observed low rates of acute toxicity, with only grade 1–2 esophagitis and diarrhea reported in 2% of the cases. Therefore, SBRT can be a safe and effective treatment for patients with spinal metastases.

This study had several limitations. First, pain response was evaluated retrospectively from chart review rather than prospectively using standardized instruments such as the Brief Pain Inventory or Visual Analog Scale. Although we included only patients with clearly documented pain responses, the subjective nature of clinical records and inter-physician variability may have introduced inconsistencies. Pain assessment based on patient recall during follow-up may also be prone to bias. Moreover, the 1–3-month post-SBRT window, while clinically relevant, may not capture the full trajectory of pain improvement or late recurrence. Despite these limitations, our approach reflects real-world practice and provides meaningful insight into treatment efficacy. Second, the retrospective design carries inherent risks of selection bias and incomplete data capture, particularly for toxicity outcomes that may be underreported in routine clinical documentation. Third, several population-specific factors limit the generalizability of our findings. The marked male predominance (74.9%), largely due to high proportions of prostate and lung cancers, may have influenced lesion characteristics and the observed VCF incidence. The limited median OS of 17.3 months also introduces competing mortality risk, potentially leading to under-detection of late local recurrences and VCFs. In addition, the heterogeneous tumor histology in our cohort complicates direct comparison with other series. Future studies with more balanced populations and longer follow-up are needed. Fourth, imaging surveillance was not standardized, and some patients lacked pre- and post-SBRT MRI, which may have affected the accuracy of response and VCF assessment. Fifth, the marked attrition in patients at risk from 1 to 2 years in our Kaplan–Meier analyses may reduce the precision of long-term estimates and introduce survivor bias, as patients remaining at 2 years likely represent a more favorable subset. Accordingly, 2-year outcomes should be interpreted with caution, whereas 1-year estimates may better reflect the overall cohort. Sixth, our VCF risk factor analysis (*n* = 20 events) carries a potential risk of overfitting. To mitigate this, we restricted multivariable analysis to covariates with *p* < 0.1 in univariate testing, emphasized effect sizes over *p*-values, and included only biologically plausible variables. Validation in larger cohorts is warranted.

Despite these limitations, this study provides real-world evidence supporting the efficacy and safety of SBRT for spinal metastases in a large, consecutive patient cohort treated with contemporary techniques and dose-fractionation schemes.

We expect future studies to further investigate the prognostic factors for LPFS and risk factors for VCF, especially based on primary tumor histology. Our study demonstrated that SBRT is an effective and well-tolerated treatment for spinal metastases in terms of pain and local tumor control.

## 5. Conclusions

In this retrospective study, we evaluated the outcomes of SBRT in spinal metastases. SBRT emerges as an effective treatment for spinal metastases, achieving high rates of pain relief, local tumor control, and an acceptable risk of vertebral compression fractures.

## Figures and Tables

**Table 1:** Patient characteristics.

Variables	Number of Patients (*n* = 179)
Age (years), median (range)	63 (29–86)
Sex	
Male	134 (74.9%)
Female	45 (25.1%)
ECOG	
0	2 (1.1%)
1	145 (81.0%)
2	32 (17.9%)
BMI (kg/m^2^), median (range)	23.5 (15.8–31.8)

Abbreviations: ECOG, Eastern Cooperative Oncology Group; BMI, body mass index.

**Table 2:** Lesion characteristics.

Variables	Number of Lesions (*n* = 217)
Bone agent use	
Yes	45 (20.7%)
No	172 (79.3%)
Histology	
Breast	7 (3.2%)
NSCLC	62 (28.6%)
Ureter	5 (2.3%)
Gastrointestinal	44 (20.3%)
Renal cell carcinoma	39 (18.0%)
Melanoma	1 (0.5%)
Prostate	46 (21.2%)
Gynecological	4 (1.8%)
Sarcoma	5 (2.3%)
Endocrinal	4 (1.8%)
Pain	
Yes	166 (76.5%)
No	51 (23.5%)
Spinal location	
Cervical	20 (9.2%)
Thoracic	93 (42.9%)
Lumbar	87 (40.1%)
Sacral	17 (7.8%)
Paraspinal extension	
Yes	48 (22.1%)
No	169 (77.9%)
Baseline vertebral compression fracture	
Yes	56 (25.8%)
No	161 (74.2%)
Bone lesion	
Lytic	126 (58.1%)
Blastic	62 (28.6%)
Mixed	29 (13.3%)
Number of segments treated	
Single	130 (60.0%)
Multiple (2–8)	87 (40.0%)

Abbreviations: NSCLC, non-small cell lung cancer.

**Table 3:** Dosimetric characteristics.

Characteristics	Number of Lesions (*n* = 217)
Fraction	
1	25 (11.5%)
3	8 (3.7%)
4	40 (18.4)
5	144 (66.4%)
GTV volume in cm^3^, median (range)	19.3 (1.1–521.0)
Total prescription dose in Gy, median (range)	30.0 (16.0–40.0)
Fractional prescription dose, Gy, median (range)	6.5 (5.0–20.0)
Prescribed BED_10_, Gy, median (range)	48.0 (30.0–72.0)
SIB	
Yes	127 (58.5%)
No	90 (41.5%)
Vertebral body dose maximum, median (range)	30 (0–40)
Vertebral body dose minimum, median (range)	24 (0–35)

Abbreviations: GTV, gross tumor volume; BED, biologically effective dose; SIB, simultaneous integrated boost.

**Table 4:** Pain assessment within three months of painful spinal metastases.

Response	Number of Painful Lesions at Baseline (*n* = 166)
Complete response	61 (36.7%)
Partial response	96 (57.8%)
Progressive pain	3 (1.8%)
Stable pain	6 (3.6%)

**Table 5:** Univariate and multivariate analysis of LPFS.

Variables	Univariate			Multivariate		
	HR	95% CI	*p*-Value	HR	95% CI	*p*-Value
Histology (radioresistant vs. radiosensitive)	1.879	0.710–4.975	0.204	-	-	-
Type of lesion (lytic/mixed vs. blastic)	5.670	1.341–23.967	0.018	5.704	1.349–24.119	0.018
Paraspinal extension (yes vs. no)	2.527	1.148–5.560	0.021	1.951	1.047–5.542	0.122
Fraction (single vs. multiple)	1.347	0.462–3.928	0.586	-	-	-
Segment (multiple vs. single)	2.665	1.221–5.813	0.014	2.686	1.228–5.874	0.013
SIB (yes vs. no)	1.667	0.745–3.732	1.667	-	-	-
BED_10_ (<50Gy vs. ≥50Gy)	1.076	0.503–2.301	0.850	-	-	-

Values in the parentheses were set as the reference. Abbreviations: LPFS, local progression free survival; HR, hazard ratio; CI, confidence interval; SIB, simultaneous integrated boost; BED, biologically effective dose.

**Table 6:** Painful vertebral compression fracture incidence by fractionation Scheme.

Fractionation	Total Number	Painful VCF Events (%)	95% CI
1 fraction	21	4 (19.0)	7.7–40.0
3 fractions	6	0 (0.0)	0.0–39.0
4 fractions	33	5 (15.2)	6.7–30.9
5 fractions	119	11 (9.2)	5.2–15.8

Abbreviations: VCF, vertebral compression fracture; CI, confidence interval.

**Table 7:** Univariate and multivariate analyses of prognostic factors for painful vertebral compression fracture.

Variables	Univariate			Multivariate		
	OR	95% CI	*p*-Value	OR	95% CI	*p*-Value
Bone agent (no vs. yes)	1.560	0.524–4.644	0.425	-	-	-
BMI (<25 vs. ≥25)	2.997	0.841–10.673	0.090	2.830	0.775–10.332	0.115
Sex (female vs. male)	1.194	0.431–3.308	0.733	-	-	-
Histology (radioresistant vs. radiosensitive)	1.032	0.322–3.305	0.957	-	-	-
Type of lesion (lytic/mixed vs. blastic)	4.500	1.006–20.120	0.049	4.212	0.825–19.173	0.063
Pre-existing VB collapse (yes vs. no)	2.123	0.808–5.058	0.127	-	-	-
Paraspinal extension (yes vs. no)	1.843	0.654–5.191	0.247	-	-	-
SINS class (II or greater vs. I)	1.957	0.758–5.049	0.165	-	-	-
Fraction (single vs. multiple)	2.088	0.625–6.972	0.231	-	-	-
SIB (yes vs. no)	1.656	0.605–4.533	0.326	-	-	-
Segment (multiple vs. single)	1.156	0.437–3.058	0.770	-	-	-
BED_3_ (≥104Gy vs. <104 Gy)	2.967	1.085–8.115	0.034	2.915	1.045–8.132	0.041

Values in the parentheses were set as the reference. Abbreviations: OR, odds ratio; CI, confidence interval; BMI, body mass index; VB, vertebral body; SINS, Spinal Instability Neoplastic Score; SIB, simultaneous integrated boost; BED, biologically effective dose.

## Data Availability

Data supporting the findings of this study are available from the corresponding author upon reasonable request.
